# Solar power generation intermittency and aggregation

**DOI:** 10.1038/s41598-022-05247-2

**Published:** 2022-01-25

**Authors:** Cong Wu, Xiao-Ping Zhang, Michael Sterling

**Affiliations:** 1 Department of Energy Strategy and Planning, State Grid Energy Research Institute (SGERI), Beijing, 102209 China; 2 Birmingham Energy Institute, University of Birmingham, Birmingham, B15 2TT UK; 3 Department of Electronic, Electrical and Systems Engineering, School of Engineering, University of Birmingham, Birmingham, B15 2TT UK

**Keywords:** Energy science and technology, Engineering

## Abstract

The inherent intermittency of solar power due to diurnal and seasonal cycles has usually resulted in the need for alternative generation sources thereby increasing system operation costs. However, when solar power is spread over a large geographical area with significant time differences, the intermittency can be significantly reduced and also the electricity market balancing cost. The aim of this article is to address the fundamental scientific question on how the intermittency of solar power generation is affected by aggregation, which is of great interest in the wider power and energy community and would have profound impacts on the solar energy integration into the energy supply and Net-Zero Implementation. This article goes beyond the typical regional analysis by investigating solar power intermittency at 5 aggregation levels from a global perspective based on global 7 year hourly meteorological re-analysis data with a fine spatial resolution of ${0.25}^{\circ }\times {0.25}^{\circ } (\sim 28 \; \mathrm{km} \times 28 \; \mathrm{km})$. In the proposed assessment framework, a coefficient of variation (CV) is used to quantify solar power intermittency and hence characterize the potential benefits of wide area solar power aggregation. A duration curve is used to characterize the intermittency in terms of power availability and a probability density function is further employed to investigate the dispersion and scaling behavior of CV at different aggregation levels. The findings indicate that the CV of solar power generation of ‘Inner Mongolia’ in China drops from 129.65 to 105.65% in the level of ‘Asia’ (by 24% decrease), to 56.11% in ‘Asia-North_America’ (by 73.54% decrease) and to the smallest 43.50% in ‘Global’ (by 86.15% decrease), nearly 3.5 times of that in ‘Asia’; (b) the availability of solar power generation increases from 52.17% in Germany, to 73.30% in ‘Europe_EU_plus’, to 77.82% in ‘Europe’, to 98.59% in ‘Europe-North_America’ (80.60% in ‘Europe-Africa’, 96.90% in ‘Europe-Asia’), to 100% in ‘Global’. Finally, conclusions and recommendations are provided to support a Net-Zero strategy.

## Introduction

Driven by an international desire to reduce carbon emissions while achieving significant cost reductions, solar power has been one of the fastest growing renewable energy sources, with worldwide deployment increasing from 40 GW in 2010 to 586 GW in 2019^1,2^, a trend which is likely to be sustained in future energy systems^3,4^. Most recently, an ‘Energy Quality’ framework was defined to measure and characterize the variations of renewable power generation^5^ where power variations of renewable energy generation consist of power fluctuations and power intermittency. Power fluctuations cover short and mid-term power variations in a timescale from seconds to hours. Intermittency is deemed to cover long-term power variations in the timescale from hours and days to years. For solar energy, intermittency is normally considered more challenging than power fluctuations. The solar radiation reaching the Earth’s surface is primarily governed by the deterministic astronomical diurnal and seasonal cycles, and the optical transmissivity of clouds and aerosols following atmospheric circulation patterns^6^. Solar power will therefore show an intermittency in timescale of hours up to months due to these diurnal and seasonal cycles, adversely affecting the stability and reliability of power grids^7^. For instance, studies of the solar energy integration into the Great Britain (GB) energy system have indicated that the cost of backup capacity for solar would increase from £2.5/MWh in 2016 to £4.5/MWh by 2030, and the high penetration of solar on the system will necessitate more backup procured through the capacity market. This would also require higher capacity payments to incentivize entry as the higher levels of solar lowers daytime power prices^8^. Much has been done to accommodate high photovoltaic (PV) penetration, such as proactive curtailment^9^, energy storage^10,11^, and demand response^12^ together with taking advantage of the spatial diversity by spreading PV farms over a large geographical area^13^.

Evidence that the intuitive correlation of solar power step change at dispersed sites decreases with increasing separation distance and decreasing time intervals has been published^14–18^. Field measurements have shown that the correlation can vary significantly in the along-wind (cloud motion) and cross-wind directions^19^ and when included, above isotropic correlation can produce a good fit with the measured data at short time intervals (< 60 s)^20^. A logarithmic model directly yielding the relative variance of step changes of a clear sky index between aggregated and single locations with regard to time resolution, field side length along cloud motion, and cloud speed has been presented in^21^. At long intervals (1–30 days), a similar correlation with distance, time interval and orientation was found in^22^, but the seasonal trend from Earth’s rotation around the Sun was removed by using a clearness index as a proxy for solar insolation. Other publications show that the variability (standard deviation) of solar production profiles was lowered by the geographical dispersion of solar-PV panels across multiple locations in Ontario^23^ and that power spectral density for large PV plants have a steeper slope than for small plants as does the aggregation of more plants, based on observed generation data in Gujarat^24^. Also based on measured generation (western US), comparison of geographical smoothing from utility-scale and building-mounted PV using power spectral density was presented in^25^. Meanwhile, it was found that solar PV did not show as much geographic smoothing as was seen for aggregated wind plants^25^, demonstrating consistency with a previous study^24^. The effect of spatial smoothing and its role in decreasing solar variability (step change) was investigated based on a clear sky index^26^, which showed that a minimum 60% of power variability was suppressed in Rajasthan for time intervals of 1–15 min.

Among existing studies, different metrics are used to characterize the smoothing effects. For example, extreme values are used in^19^, variance of step changes^21^, standard deviation (STD) of the step change/ramping rate^14,16–18,22,26^, STD of the production^23^, and Fourier transform estimates of the power spectral density (PSD)^15,24,25^. Timescales (durations) considered are mainly minutes^16,19–21^, hours^14,17,18,26^, months^23^ and years^15,24,25^. Furthermore, the geographical scale for solar power aggregation varies with plant/site^16,19–21,27^, to state^15,18,23,24,26^ and to sub-region^14,25^ but with a limited number of PV sites/stations. Most existing studies have been focused on the impact of solar power aggregation on short and mid-term power variations in terms of fluctuations rather than long term variations in terms of intermittency. Furthermore, most studies have investigated the smoothing effects for aggregation within a country and levels below and usually a limited number of PV sites/stations are aggregated. It is well recognized internationally that the intermittency of solar energy is a fundamental technical/economic barrier which limits the penetration level of solar power in the energy supply.

This article goes beyond a regional scale to consider global solar energy aggregation at 5 different levels/scales, and hence quantifies the impacts of aggregation across these levels/scales on the intermittency of solar power generation in terms of three metrics, namely, coefficient of variation (CV), duration curve and probability density function (PDF) of CV. The elements of geographical scale at each level are shown in Tables S1–S3 in Supplementary Information. The approach adopted in this article is:

1. To propose a five level hierarchy of geographical scale for solar power aggregation, spanning from state (or equivalent province/country), to region, continent, inter-continent, and up to global.
2. To generate detailed solar power series for states worldwide using global hourly meteorological re-analysis data during 2011–2017 with spatial resolution of ${0.25}^{\circ }\times {0.25}^{\circ }$ (approximately $28\; \mathrm{ km}\times 28 \; \mathrm{ km}$). Specifically, 50% of the grid cells with the highest 7 year average capacity factor are selected, sorted, and grouped with an interval of 10%, and the weighted sum of these five groups is treated as the equivalent solar power in a state. The solar power series of an area at a higher level is aggregated from the power of its sub-areas, taking the resource potential as the weight.
3. To propose two assessment metrics to quantify the solar power intermittency at different levels. A coefficient of variation (CV) is used to measure the 7-year variability of solar power series and a duration curve is utilized to quantify the availability of solar power during that 7 years.

## Results

### Solar power series and capacity factors

**Figure 1:**
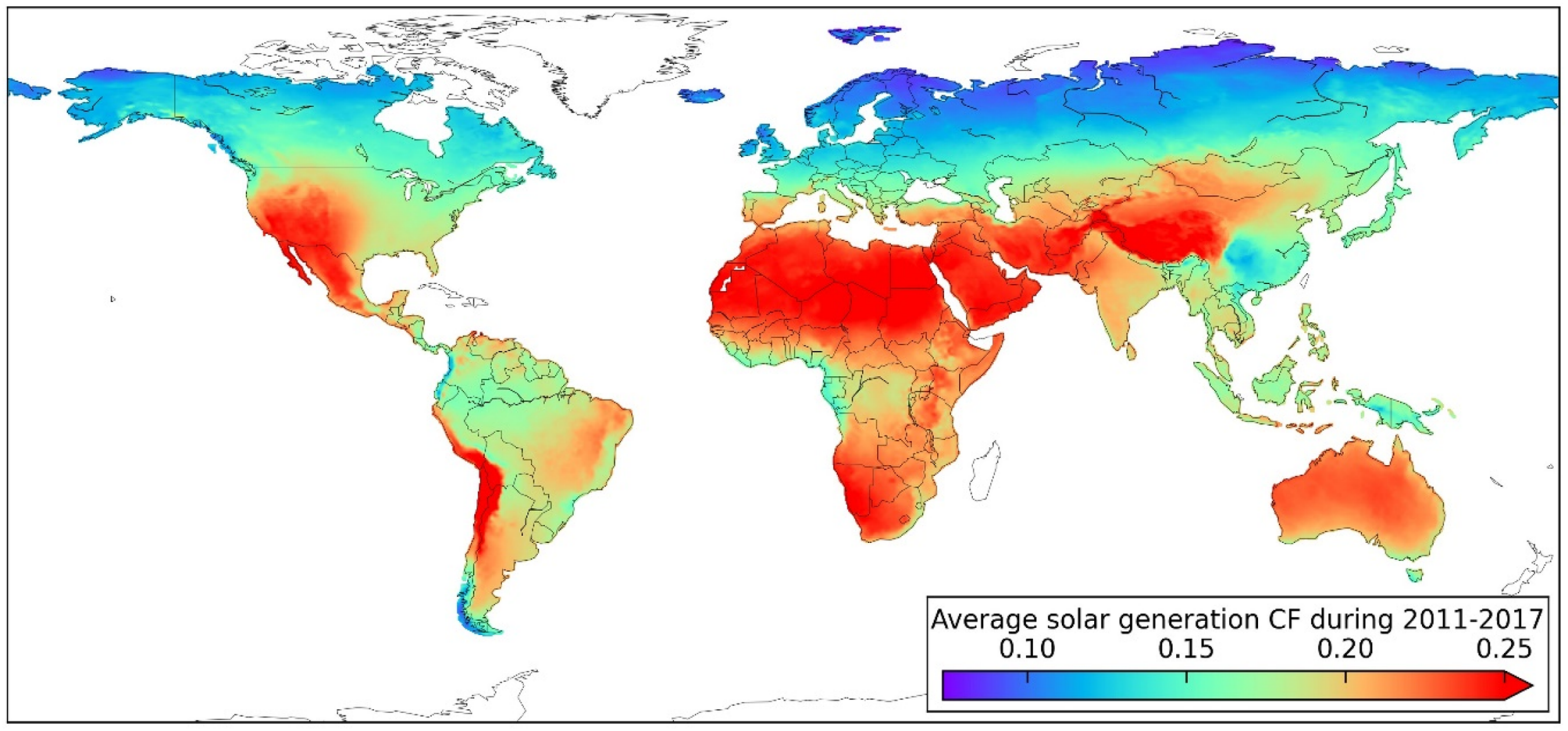
Global capacity factors of solar generation (the figure was generated with Python 3.8.2 https://www.python.org/downloads/release/python-382/).

The average capacity factors for solar generation globally during 2011–2017 are shown in Fig. 1 based on 224,750 grid cells. The potential capacity and average capacity factor of regions, continents, inter-continents, and the global as well as for some typical countries/states are shown in Tables S4 and S5 in Supplementary Information.

The south-western part of North America, Africa, West Asia, and south-western China have higher CFs from a global perspective. It is obvious that the zones close to the Poles have lower CFs. Moreover, it is noted that the CFs of areas along the equator are relatively lower than for adjacent zones because of a tropical rainforest climate with frequent rainfall and thus solar irradiance reaching the earth land surface will be greatly weakened by cloud.

**Figure 2:**
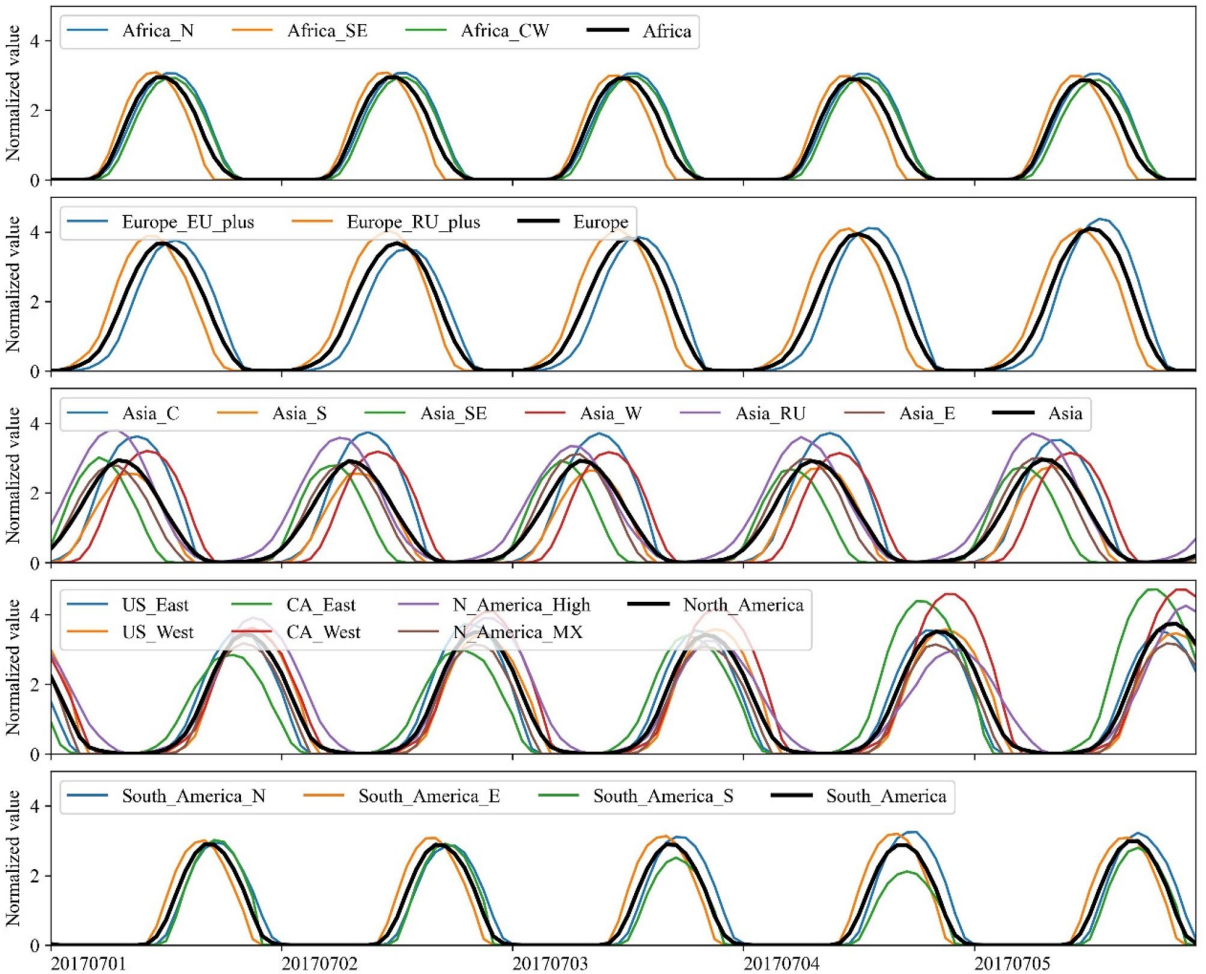
Solar power time series (1st–5th July 2017) of regions in each continent.

**Figure 3:**
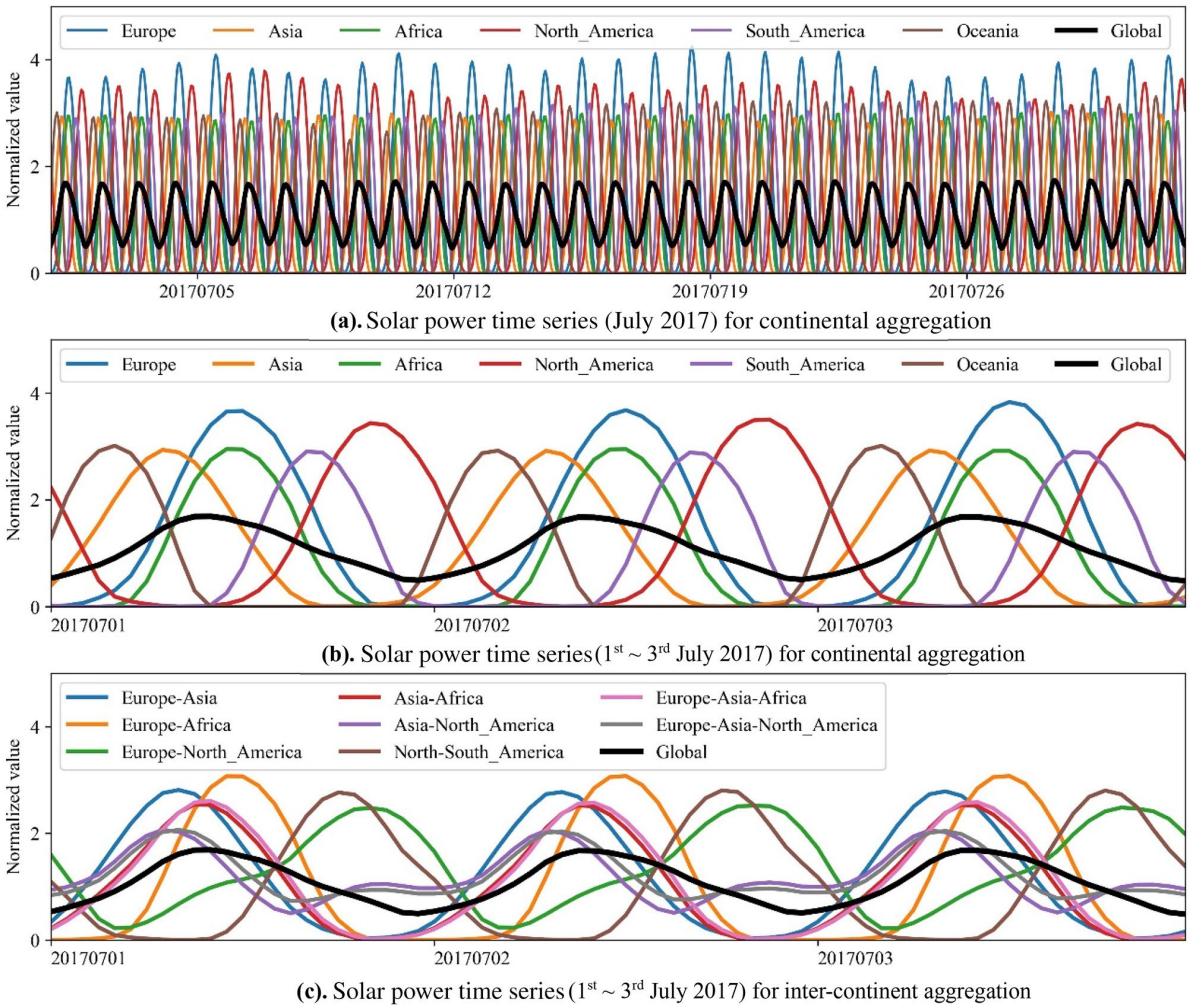
Solar power time series of continental and inter-continental aggregation.

A segment of solar power series in summer (Northern Hemisphere) for aggregation at region and continent level is illustrated in Fig. 2 and that for aggregation at inter-continent and global level is shown in Fig. 3. Those in winter (Northern Hemisphere) are shown in Figs. S1 and S2 in Supplementary Information, respectively. It should be mentioned that the solar power series is normalized by being divided by its average. Tables S1–S3 in Supplementary Information show the detailed definition of the five level hierarchy with geographical details.

As in Figs. 2 and S1, when regional solar power series are aggregated at continent level, the normalized peak values become slightly lower, especially for ‘Asia’ and ‘North_America’ that comprise several regions. This illustrates that continental solar power becomes more uniform than that of an individual region. However, there are some regions whose normalized power profile seems to be more stable than that of a continent, e.g., ‘South_America_N’ in Fig. 2 and ‘Asia_RU’ in Fig. S1. The profiles in these figures are just a 5-day segment out of 7-year series so from a 7 year perspective, continental aggregation actually performs better than regional aggregation in terms of lower CV as shown in Table S5 in Supplementary Information.

Additionally, it is noted that there are small time differences between the regions within the same continent, especially for ‘Africa’ and ‘South_America’, and it is expected that those between the countries/states/provinces will be fewer though not shown here. As a result, the inherent intermittency of solar power is still strong for each continent but the duration time with non-zero outputs is longer than that of individual regional solar power series.

In contrast, as in Fig. 3b, the time differences between some continents are large, e.g., up to 13 h between ‘Asia’ and ‘North_America’. The time difference between ‘Europe’ and ‘North_America’, ‘Europe’ and ‘Asia’, ‘North_America’ and ‘South_America’ is around 8 h, 5 h, and 4 h, respectively, while there is nearly no time difference between ‘Europe’ and ‘Africa’. Therefore, it is shown in Fig. 3c that in summer (Northern Hemisphere) the solar power aggregation of ‘Europe-North_America’, and in particular ‘Asia-North_America’ will eliminate the zero-output periods and smooth the power series with a decreasing peak-to-peak (PTP) value and is better than that of ‘Europe-Africa’. However, in winter, there are still periods with zero outputs for ‘Europe-North_America’, as shown in Fig. S2c. Moreover, the profiles of ‘Asia-Africa’ and ‘Asia-North_America’ are influenced to a small extent after ‘Europe’ joins the combination as a result of the relatively low potential capacity in ‘Europe’ (as shown in Table S5). The solar power aggregation of multiple continents in the close proximity (e.g., ‘Europe-Asia-Africa’) still show the intermittency of solar power but the periods with zero outputs become shorter. The inter-continental aggregation between ‘Asia’ and ‘North_America’ performs much better than the other inter-continental combinations.

It is therefore clear that the global aggregation of solar power of all continents together can lead to smoother power series together with lower PTP value, and shorter, shallower troughs in power profiles.

### Coefficient of variation of power series at 5 different aggregation levels

**Figure 4:**
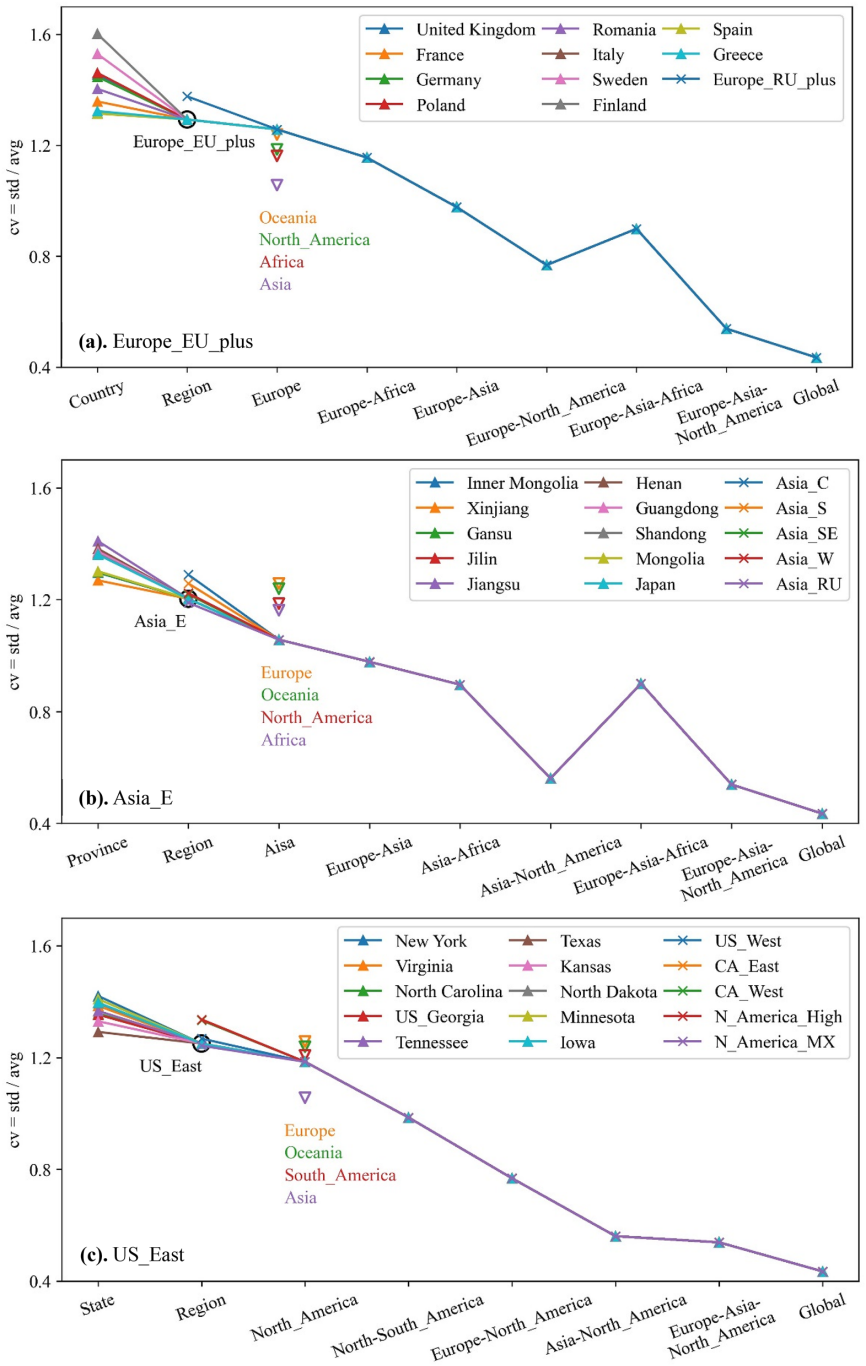
CV of 7-year solar power series for all regions at different aggregation levels.

The varying trends in the coefficient of variation (CV) of solar power series at different aggregation levels are illustrated in Fig. 4, where for region ‘Europe_EU_plus’, ‘Asia_E’ and ‘US_East’ (marked by ∆), it is shown from the lowest country level but for other regions (marked by ‘x’) for clarity it is shown from regional level. The basic data for plotting are shown in Tables S4 and S5 in Supplementary Information.

- As shown in Fig. 4, the CV of solar power decreases continuously with the increasing aggregation scale from the lowest country level to regional and continent level. Specifically, regional aggregation will decrease the CV for 97.8% of the global countries/provinces/states and continental aggregation will decrease the CV for all the countries/provinces/states, as in Tables S6 and S7 in Supplementary Information. Although the solar power aggregated in a continent spanning wider time zones has smaller CV, such as ‘Asia’, the overall CV reduction by up to continental aggregation (from ‘Country’ level) is relatively small compared to that by global aggregation and some inter-continent aggregation scenarios. For example, the CV of ‘Inner Mongolia’ in China drops from 129.65 to 105.65%, at the level of ‘Asia’ (by 24% decrease), to 56.11%, at the level of ‘Asia-North_America’ (by 73.54% decrease) and to the smallest 43.50%, at the level of ‘Global’ (by 86.15% decrease), nearly 3.5 times of that at the level ‘Asia’.
- Furthermore, any inter-continent aggregation can yield a further reduction of the CV compared to individual continental aggregation, but the amount varies, depending largely on the time differences between the continents. As shown in Fig. 4a,b, aggregation of ‘Europe-North_America’ with 8-h difference and that of ‘Asia-North_America’ with 13-h difference performs respectively much better than other two continent aggregation scenarios. The CV of ‘Africa’ decreases very slightly (less than 1%) after ‘Africa’ aggregates with ‘Europe’ while that of ‘Asia’ decreases approximately by 8%, both with much larger average solar power (the product of potential capacity and capacity factor) than that of ‘Europe’. This supports the intuitive view that areas locating in similar time zones lack complementarity effects and hence aggregation benefits. Besides, it is noted that ‘Europe-Asia-Africa’ performs comparably with ‘Asia-Africa’ because of the lack of time difference between ‘Europe’ and ‘Africa’ and lower average solar power in ‘Europe’.
- In addition, global aggregation of solar power of all continents will introduce the least CV because solar power from diverse time zones are complementary and the intermittency can be greatly decreased. The overall potential capacity in ‘Asia’, ‘Africa’, and ‘Europe’ is larger than that of ‘North_America’, with the result that the global power series fluctuate roughly sinusoidally with a constant component as shown in Fig. 3b. One possible solution to smooth the global profile and decrease the global CV would be to increase the capacity ratio of ‘North_America’. It could be expected that with optimal capacity allocation in each continent, region, or country, the CV of globally aggregated solar power can therefore be much lower.

### Duration curves at different aggregation levels

**Figure 5:**
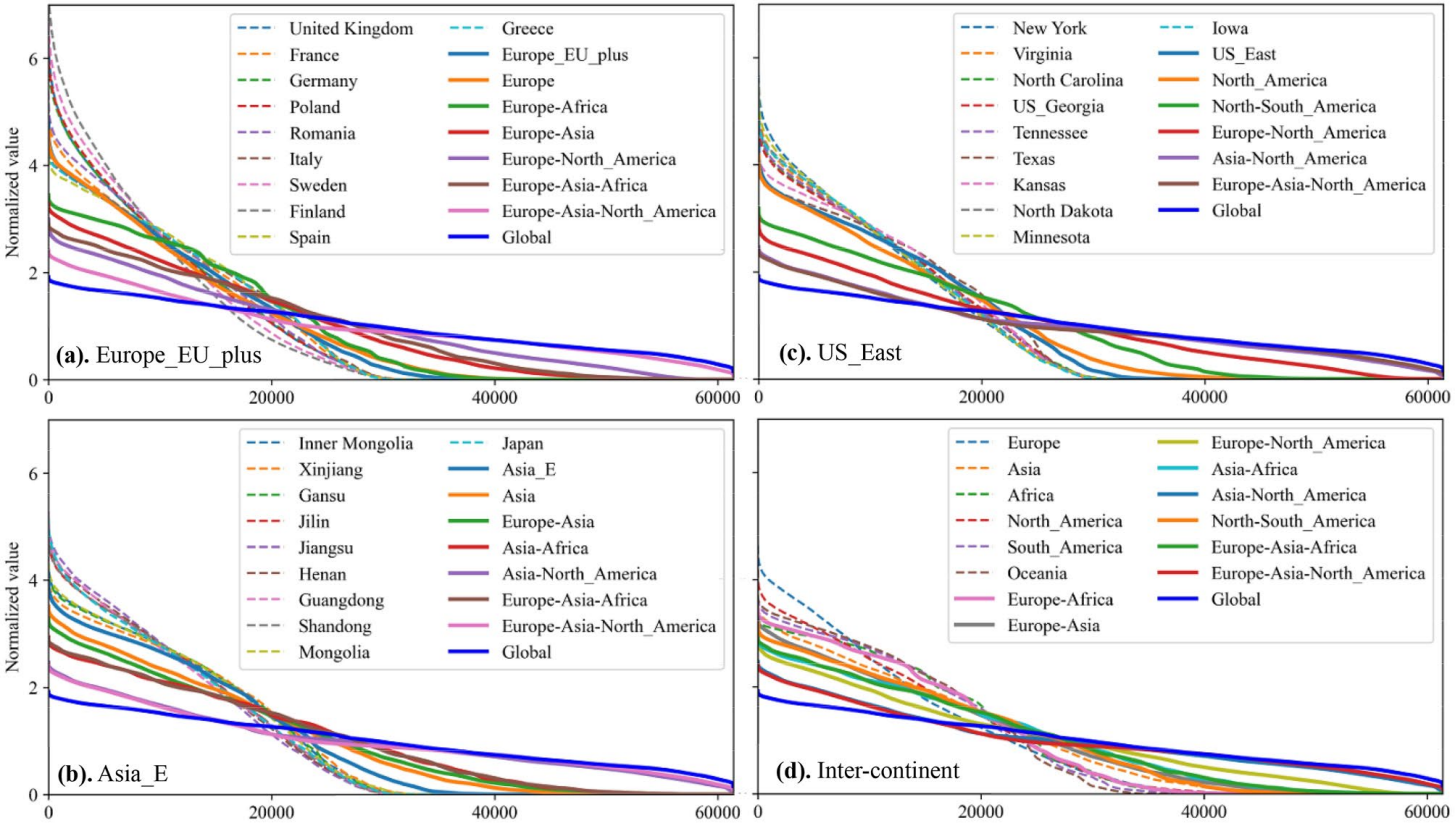
Duration curve for three typical regions at different aggregation levels and various inter-continent aggregations.

The duration curves of 7 year solar power aggregated at different levels for three typical regions are shown in Fig. 5 where the dashed lines represent the duration curves at lowest level in each region. Power availabilities for various aggregation scenarios are shown in Tables S4 and S5 in Supplementary Information.

As shown in Fig. 5, several trends can be found as the aggregation scale of solar power increases from country to final global level:

- First, the availability of power, i.e., total hours with non-zero outputs during 7 years, increases continuously. In Tables S4 and S5, for example, the availability increases from 52.17% in Germany (32,016 out of 613,687 h during 2011–2017), to 73.30% in ‘Europe_EU_plus’, to 77.82% in ‘Europe’, to 98.59% in ‘Europe-North_America’ (80.60% in ‘Europe-Africa’, 96.90% in ‘Europe-Asia’), to 100% in ‘Global’.
- There is still unavailability for solar power aggregated between continents with time differences of 8 h or less, such as ‘North-South_America’ (4 h), ‘Europe-Asia’ (5 h), and ‘Europe-North_America’ (8 h). In contrast, for continents with a longer time difference, for instance, ‘Asia’ and ‘North_America’ (13 h), the aggregation can significantly alleviate the intermittency of solar power and lead to full power availability all year round.
- Another trend is that the duration curves become flatter as aggregation is scaled up. It is obvious that the extremely high values (normalized) at regional level are much lower than that at country level and will decrease continuously. Aggregation of solar power at a higher level makes the duration flatten along the horizontal axis. Meanwhile, although ‘Asia-North_America’, ‘Europe-Asia-North_America’, and ‘Global’ have non-zero outputs all the time, the duration curve of ‘Global’ is obviously smoother than the others as in Fig. 5c because of the complementary effects of solar generation from diverse time zones.

### Probability density function of CV at different aggregation levels

**Figure 6:**
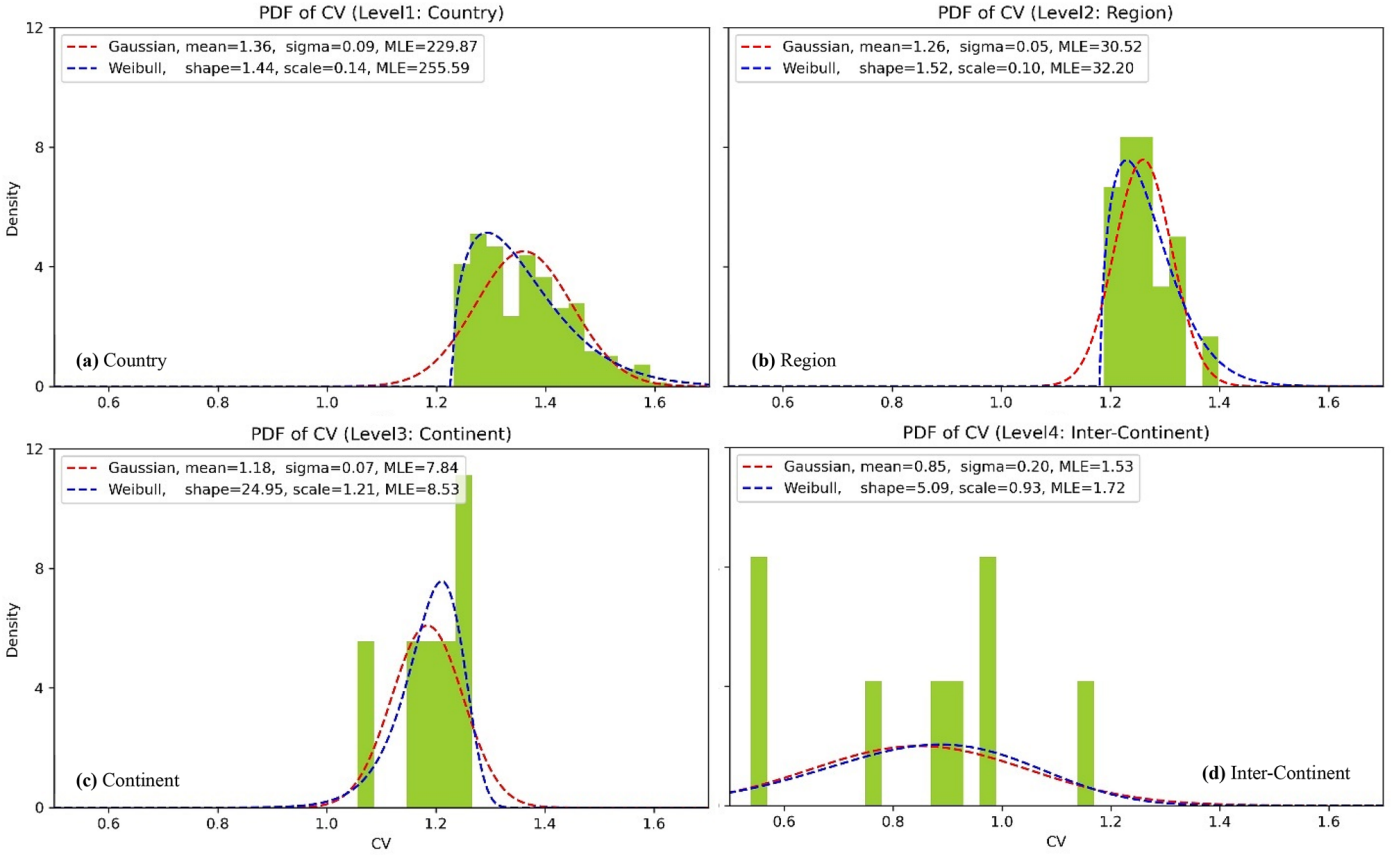
Power density function (PDF) of CV at different aggregation levels.

The probability density functions (PDF) of CV at different aggregation levels are shown in Fig. 6, where ‘mean’ and ‘sigma’ are the parameters for fitted Gaussian distribution, ‘shape’ and ‘scale’ are the parameters for fitted Weibull distribution, and MLE means Maximum Likelihood Estimation (log-likelihood in this study). Log-likelihood is the logarithm of the probability that a given set of observations is observed given a probability distribution.

As shown in Fig. 6, two major phenomena can be found as follows.

- Weibull distribution is more fitted with the samples/observations of CV than Gaussian distribution at all aggregations levels in terms of MLE, e.g., in Fig. 6a, the MLE of Weibull distribution is 255.59 and higher than that of Gaussian distribution (229.87). This suggests that the dispersion of CV at each level is generally subject to Weibull distribution.
- With the aggregation level being increased, there are significantly fewer samples, e.g., there are six continents to be considered in this study and thus six samples at level 3—‘Continent’, as a result, the PDF of CV at this level can hardly be fitted as well as that at levels of ‘Country’ and ‘Region’. Additionally, the CV is generally smaller with increasing aggregation level and thus wider geographical scale, which supports the conclusions in previous sections.

## Conclusions and recommendations

This article has investigated the complementary effects of solar power aggregation at five levels from a low level of state/province/country to a high of global level. The impacts of geographical aggregation at these 5-diffeernt levels on the intermittency of solar energy have been quantified in terms of two metrics, namely, coefficient of variation and duration curve. A third index, namely the probability density function is further employed to investigate the dispersion and scaling behavior of CV at different aggregation levels. Seven year (2011–2017) hourly meteorological re-analysis data with a spatial resolution of ${0.25}^{\circ }\times {0.25}^{\circ }$ (approximately $28\; \mathrm{ km}\times 28 \; \mathrm{km}$) has been converted into solar power series globally, so there are a total of 224,750 grid cells and 5 TB of data. The weighted sum of solar power series from 50% of the grid cells with highest average capacity factor within a state/province/country has been taken as the equivalent solar power in this area.

It is therefore clear that:

- The CV of solar power decreases continuously with the increasing aggregation scale from the lowest country level to regional and continent level. Although the solar power aggregated in a continent spanning wider time zones has smaller CV, such as ‘Asia’, the overall CV reduction by up to continental aggregation (from ‘Country’ level) is smaller than that by global aggregation and some inter-continent aggregations. For example, the CV of ‘Inner Mongolia’ in China drops from 129.65 to 105.65%, at the level of ‘Asia’ (by 24% decrease), to 56.11%, at the level of ‘Asia-North_America’ (by 73.54% decrease) and to the smallest 43.50%, at the level of ‘Global’ (by 86.15% decrease), nearly 3.5 times of that at the level ‘Asia’.
- Any inter-continent aggregation can yield a further reduction of the CV compared to individual continental aggregation, but the amount varies, depending largely on the time differences between the continents. Basically, longer time differences result in smaller CV. For instance, aggregation of ‘Europe-North_America’ with 8-h difference and that of ‘Asia-North_America’ with 13-h difference perform, respectively, much better than the other related inter-continental aggregation scenarios confirming, from actual data, an intuitive view. Global aggregation will introduce the least CV because solar power from diverse time zones assembles complementarily, indicating the intermittency can be greatly decreased.
- With increasing aggregation scale, the availability of power in terms of the duration curve, i.e., total hours with non-zero outputs during 7 years, increases continuously. For example, the availability increases from 52.17% in Germany (32,016 out of 613,687 h during 2011–2017), to 73.30% in ‘Europe_EU_plus’, to 77.82% in ‘Europe’, to 98.59% in ‘Europe-North_America’ (80.60% in ‘Europe-Africa’, 96.90% in ‘Europe-Asia’), to 100% in ‘Global’. However, there is still unavailability for solar power aggregated between continents with time differences of 8 h or less, such as ‘North-South_America’ (4 h), and ‘Europe-North_America’ (8 h). In contrast, the aggregation of ‘Asia-North_America’ with 13-h difference will significantly alleviate the intermittency of solar power and lead to full power availability all year round.
- Duration curves become flatter as the aggregation is scaled up. The extreme high values (normalized) decrease continuously. Although both ‘Europe-Asia-North_America’ and ‘Global’ have non-zero outputs all the time, the duration curve of ‘Global’ is obviously smoother than that of ‘Europe-Asia-North_America’.
- Weibull distribution is more fitted with the samples of CV than Gaussian distribution at all aggregations levels in terms of MLE. With the increasing aggregation level, the fitted distributions of the PDF do not perform as well as that at levels of ‘Country’ and ‘Region’ due to limited samples of CV at higher levels.
- The general findings for solar energy integration within different geographical scales using the quantifiable metrics provide useful insights for policy makers into the cooperation opportunities at different levels/scales up to global level in the context of a Net-Zero target implementation. Moreover, the research indicates where potential interconnections might bring significant economic and operational benefits and facilitates further study of the costs of long distance links against the potential savings.

## Methods and data

### Hierarchy of geographical scale

Five aggregation levels are proposed to investigate the complementary effects of solar power in this study, i.e., ‘Country/Province/State’, ‘Region’, ‘Inter-continent’, ‘Continent’ and ‘Global’. The geographical scale of the elements at each level are shown in detail in Tables S1–S3 in Supplementary Information.

#### ‘Country/Province/State’ (Level 1)

It is the lowest level within the hierarchy and the name may differ worldwide, e.g., ‘Country’ for European Union (EU), ‘Province’ for China and ‘State’ for the United States (US).

#### ‘Region’ (Level 2)

Solar power series in several ‘Country/Province/State’ in proximity to each other are further aggregated into a ‘Region’ which is the sub-area of a ‘Continent’.

#### ‘Continent’ (Level 3)

One ‘Continent’ is normally made up of several ‘Regions’. It refers to six continents in this study, namely Europe, Asia, Africa, North America, South America, and Oceania. The solar power series will be aggregated at each continental level/scale, respectively.

#### ‘Inter-continent’ (Level 4)

To aggregate with adjacent continent provides wider scale. Solar power in at most three continents will be aggregated. Specifically, various aggregation scenarios based on two continents and two scenarios of ‘Europe-Asia-Africa’ and ‘Europe-Asia-North_America’ are considered.

#### ‘Global’ (Level 5)

It includes all the major countries worldwide on seven continents except ‘Antarctica’, and is the highest level within the hierarchy for aggregation with the largest geographical scale.

Three regions (EU, Asia East, and US East) of particular interest are exemplified to describe the hierarchy. Higher than country, ‘Germany’ is in region ‘Europe_EU_plus’ that comprises most EU countries plus several adjacent countries, e.g., Norway, Serbia and Albania; the remaining levels are ‘Europe’, ‘Europe-Africa’ and ‘Global’. Similarly, the hierarchy for Asia East is: ‘Jiangsu’ (a province in China) at level 1, ‘Asia_E’ (eastern Asia) at level 2, ‘Asia’ at level 3, ‘Europe-Asia’ at level 4, and ‘Global’ at level 5 – the highest level. The hierarchy for US East is: ‘New Jersey’ (a state in the US) at level 1, ‘US_East’ (eastern US) at level 2, ‘North America’ at level 3, ‘North–South America’ at level 4 and ‘Global’ at level 5. It should be noted that ‘Country’ is the minimum scale except for Russia, Canada, China, and the US.

### Hourly solar power series during 2011–2017

In this study, historical meteorological re-analysis data of up to 7 years with spatial resolution (longitude × latitude) of ${0.25}^{\circ }\times {0.25}^{\circ }$ (approximately $28 \; \mathrm{ km}\times 28 \; \mathrm{km}$) and temporal resolution of one hour are converted into solar power series based on a similar method to that proposed in^28^ and widely applied in^29–32^.

There are a total of 224,750 grid cells and the meteorological data takes up a storage space of some 5 Terabytes (TB). Due to the large amounts of data involved, it took more than a month to download the 7-year hourly weather data because of variable international network speeds, website response times and computer hardware capabilities. A further 90 h were required to convert the weather data downloaded into solar power series. All the data analysis is coded with Python on an IntelCore-i5-8300H/2.3 GHz personal laptop with 8 G memory. Also, all the figures/images are generated with Python 3.8.2^33^ using the same laptop.

#### Weather datasets

A dataset called ‘ERA5’ is employed in this study, which is produced by the European Centre for Medium-Range Weather Forecasts (ECMWF)^34^. The geographical definition of administrative boundaries are retrieved from the Natural Earth Dataset^35^ for country shapes and the Database of Global Administrative Areas (GADM)^36^ provides different layers of boundaries within each country.

#### Converting model

A CdTe-based PV model with fixed tilt angle optimized by the grid cell’s latitude is chosen to generate solar power series. An optimal tilt angle for the given latitude is obtained using a simple method^37^ which works for latitudes between 0 and 50 and returns a static 40° angle for higher latitudes where the angle may not be that important^38,39^. The fitted model of CdTe solar panel was presented by Huld^40^ to estimate the energy yield of PV modules based on irradiance and temperature. This function in ‘Atlite’ is adapted from another python package called ‘GSEE’^41^. See^31^ for more details about solar converting models.

#### Converting and aggregating at level ‘Country/Province/State’

It is assumed that an equivalent 1-MW PV panels will be placed at the center of each raster cell to represent the solar generation of the cell. Then the 7 year solar power series is converted from the weather data for each cell and the corresponding average capacity factor (CF) is also calculated. Based on the CF, 50% of the grid cells within a ‘Country/Province/State’ that has the highest average CF are selected, sorted and aggregated into 5 groups with an interval of 10%, which indicates the layout of PV sites within a ‘Country/Province/State’ spreads a half of the overall geographical scale.

It is assumed that the top 0–10% and 10–20% of the raster cells with highest CF are weighted by 0.3, 20–30% of the cells are weighted by 0.2, and last 30–40% and 40–50% of the cells are weighted by 0.1^42^, which leads to a total of five groups. Therefore, the final solar power series expressed as CF at lowest level ‘Country/Province/State’ will be generated.

1 $${cf}_{t}=\sum_{i=1}^{5}{w}_{i} \left (\frac{{p}_{i,t}}{{c}_{i}} \right) t\in \left[1,{T}_{max}\right]$$

where ${p}_{i,t}$ is the aggregated power of group *i* at hour *t*, ${c}_{i}$ is the corresponding aggregated capacity within a country/province/state and thus $(\frac{{p}_{i,t}}{{c}_{i}})$ denotes the solar power CF of group *i* at hour *t*. ${w}_{i}$ is the weight of group *i* (e.g., 0.3 for the group with top 0–10% raster cells) and ${cf}_{t}$ is the final equivalent CF series of solar power at level of ‘Country/Province/State’, which is the weighted sum of the CF series of all five groups within this country/province/state. ${T}_{max}=61320$ denotes the total hours for 7 years (2011–2017).

#### Potential capacity at level ‘Country/Province/State’

Within a ‘Country/Province/State’, it is assumed that only 6% of the land area can be covered by PV plants^43^. The installation density for PV cells is assumed to be 81.8 MW/km^2^^44^.

2 $$Cap=(\alpha S)\rho$$

where $S$ and $\alpha$ denote the total land area (km^2^) of a country/province/state, the ratio (6% in this study as introduced previously) of available area for installing PV plants, and thus $(\alpha S)$ denotes the available area for installing PV plants. $\rho$ is the installation density (MW/km^2^) and $Cap$ is the calculated capacity of solar generation in this country/province/state.

#### Aggregating at higher levels

The solar power series expressed as CF and the installing potential for any higher level than ‘Country/Province/State’ is aggregated from the solar power series at its lower level. The capacity (${Cap}^{n}$) of a certain area is calculated as the sum of the capacities of all the sub-areas (${Cap}_{j}^{n-1}$) within this area; the equivalent CF series (${cf}_{t}^{n}$) of a certain area is calculated as the weighted sum of the solar power series in sub-areas *j* (${cf}_{j,t}^{n-1}$), where the ratio between potential capacities of sub-area and area is considered to be the weight, i.e., $(\frac{{Cap}_{j}^{n-1}}{{Cap}^{n}})$.

3 $$\left\{\begin{array}{l}{Cap}^{n}=\sum_{j}{Cap}_{j}^{n-1}\\ {cf}_{t}^{n}=\sum_{j}{cf}_{j,t}^{n-1} \left (\frac{{Cap}_{j}^{n-1}}{{Cap}^{n}} \right) t\in \left[1,{T}_{max}\right]\end{array}\right.$$

### Metrics for characterizing intermittency

In this paper, two metrics are used to assess the intermittency of solar power generation, which are detailed as follows.

#### Coefficient of variation of 7 year solar power series

The statistical index, Standard Deviation (STD), is usually used to measure the dispersion of a dataset relative to its mean. To compare the degree of variation for solar power series at different aggregation scale, the coefficient of variation (CV), also known as relative STD, is used in this paper. It should be noted that solar power is expressed as nominal value, calculated based on the capacity factor and potential capacity obtained in the previous section.

4 $$\left\{\begin{array}{l}{{p}_{t}}=cf_{t}Cap \,\,\,\,\,\,\,\,t\in \left[1,{T}_{max}\right]\\ \sigma =\sqrt{\frac{1}{{T}_{max}}\sum_{i=1}^{{T}_{max}}{\left({p}_{t}-\mu \right)}^{2}}\\ \varphi =\sigma /\mu =\sqrt{\frac{1}{{T}_{max}}\sum_{i=1}^{{T}_{max}}{({p}_{t}-\mu )}^{2}}/\mu \times 100\%\end{array}\right.$$

where $\varphi$, $\sigma$ and $\mu$ are the CV, STD and mean of the 7 year solar power series, respectively, of a certain area at a particular level. ${cf}_{t}$ is defined in (1). $Cap$ is defined in (2).

#### Duration curve

The duration curve, which is defined as the solar power time series sorted in descending order, is another effective approach for characterizing the intermittency of solar power time series in terms of availability. In order to compare the duration curves of solar power time series aggregated at different levels, each solar power duration curve of the 7 year solar power series of a certain area is therefore standardized based on its 7 year average as follows.

5 $${p}_{t}^{{\prime}}=\frac{{p}_{t}}{\mu }\quad t\in \left[1,{T}_{max}\right]$$

#### Probability density function of CV at different aggregation levels

In order to explore the dispersion and scaling behavior of CV at different aggregation levels, the probability density function (PDF) of CV is further employed as a third index. Additionally, each PDF is fitted with Gaussian and Weibull distribution respectively in this study based on Maximum Likelihood Estimation (MLE) which is normally used to estimate the parameters of a distribution based on observed samples. Specifically, the fit is computed by minimizing the negative log-likelihood function through Scipy Package in Python^45^. It is worth noting that due to very limited samples at highest level (Level 5—Global, only 1 sample), the PDF is not plotted for this level.

## Supplementary Information

Supplementary Information.
