# Supplementary material for: Solar power generation intermittency and aggregation
Source: Sci Rep. 2022 Jan 25;12:1363. doi: 10.1038/s41598-022-05247-2 (PMC8789829; doi:10.1038/s41598-022-05247-2)
Supplement: Supplementary file 1 — Supplementary Information. [file 41598_2022_5247_MOESM1_ESM.docx]

**Solar Power Generation Intermittency and Aggregation**

Cong Wu^1,2^, Xiao-Ping Zhang^1,2,*^, Michael Sterling^1,2^

^1^Birmingham Energy Institute, University of Birmingham, Birmingham B15 2TT, U.K. ^2^Department of Electronic, Electrical and Systems Engineering, School of Engineering, University of Birmingham, Birmingham B15 2TT, U.K.

^*^Corresponding author: Xiao-Ping Zhang, email: [X.P.Zhang@bham.ac.uk](mailto:X.P.Zhang@bham.ac.uk)

**Supplementary Information**

**Table S2.** Geographical scale of regions and continents

| Region/Continent | Elements |
| --- | --- |
| US_East | US_NENY, US_Mid_Atlantic, US_Carolinas, US_South, US_Tennessee, US_Midwest, US_Central, US_Texas |
| US_West | US_Southwest, US_Northwest, US_California |
| CA_East | 'Ontario', 'Quebec', 'New Brunswick', 'Prince Edward Island', 'Nova Scotia', 'Newfoundland and Labrador' |
| CA_West | 'Manitoba', 'Saskatchewan', 'Alberta', 'British Columbia' |
| N_America_H | 'USA_Alaska', 'CA_Yukon', 'CA_Northwest', 'CA_Nunavut' |
| N_America_MX | 'Mexico' |
| Asia_E | CN_Northwest, CN_North, CN_Northeast, CN_East, CN_Central, CN_South, CN_Tibet, Asia_Mongolia, Japan_Korea |
| Asia_C | 'Kazakhstan', 'Uzbekistan', 'Tajikistan', 'Kyrgyzstan', 'Turkmenistan', 'Afghanistan' |
| Asia_S | 'India', 'Pakistan', 'Bangladesh', 'Bhutan', 'Nepal', 'Sri Lanka' |
| Asia_SE | 'Cambodia', 'Thailand', 'Laos', 'Myanmar', 'Vietnam', 'Philippines', 'Malaysia', 'Indonesia' (merge with ‘Timor-Leste’, ‘Brunei’, and ‘Papua New Guinea’) |
| Asia_W | 'Iran', 'Iraq', 'Israel', 'Jordan', 'Kuwait', 'Lebanon', 'Oman', 'Qatar', 'Saudi Arabia', 'Syria', 'United Arab Emirates', 'Yemen', 'Turkey', 'Palestine' |
| Asia_RU | 'RU_Siberian_W', 'RU_Siberian_E', 'RU_Siberian_N', 'RU_FarEastern_NW', 'RU_FarEastern_L', 'RU_FarEastern_U' |
| Europe_EU_plus | Iberia, British_Isles, Europe_CW, Nordic_Baltic, Europe_E, Europe_S |
| Europe_RU_Plus | 'RU_Central', 'RU_Volga', 'RU_Northwestern_E', 'RU_Northwestern_W', 'RU_Southern_Caucasian', 'RU_Ural_N', 'RU_Ural_S', 'Ukraine', 'Belarus', 'Georgia' |
| Africa_N | 'Egypt', 'Libya', 'Algeria', 'Morocco', 'Tunisia' |
| Africa_SE | 'Dem. Rep. Congo', 'Angola', 'Zimbabwe', 'Botswana', 'Namibia', 'Zambia', 'Mozambique' (merge with Malawi), 'South Africa' (merge with ‘eSwatini’, ’Lesotho’), 'Tanzania' (merge with ‘Burundi’, ‘Rwanda’), 'Somalia', 'Sudan', 'S. Sudan', 'Ethiopia' (merge with ‘Djibouti’, ‘Eritrea’), 'Kenya' (merge with ‘Uganda’) |
| Africa_CW | 'Chad', 'Cameroon', 'Congo' (merge with ‘Gabon’, ‘Eq.Guinea’), 'Central African Rep.', 'Mali', 'Mauritania', 'Niger', 'Nigeria', 'Guinea' (merge with ‘Senegal’, ‘Gambia’, ‘Guinea-Bissau’, ‘Sierra Leone’, ‘Liberia’), 'Ghana' (merge with ‘Togo’, ‘Benin’, ‘Burkina Faso’, ‘Cote d’lovire’) |
| South_America_N | 'Guatemala' (merge with ‘Belize’), 'Honduras' (merge with 'El Salvador', ‘Nicaragua’), 'Panama' (merge with ‘Costa_Rica’), 'Colombia', 'Venezuela', 'Guyana', 'Guyane', 'Suriname', 'Ecuador', 'Peru', 'Bolivia' |
| South_America_E | 'Brazil' |
| South_America_S | 'Chile', 'Argentina', 'Uruguay', 'Paraguay' |
| Europe | Iberia, British_Isles, Europe_CW, Nordic_Baltic, Europe_E, Europe_S, Europe_RU_Plus |
| Asia | Asia_E, Asia_C, Asia_S, Asia_SE, Asia_W, Asia_RU |
| Africa | Africa_N, Africa_SE, Africa_CW |
| North_America | US_East, US_West, CA_West, CA_East, N_America_H, N_America_MX |
| South_America | South_America_N, South_America_E, South_America_S |
| Oceania | ‘Australia’ |
| Global | Europe, Asia, Africa, North_America, South_America, Oceania |

Note: 1) Shapefiles for elements in Russia are manually generated according to the division of federal districts along with nearly 85 subjects based on ‘level 1’ shapefiles of Russia, see Table S3. 2) Geographical scale of inter-continent scenarios is not shown here, which can be identified directly according to its name. 3) This study takes no position on any sovereign status and boundary delimitation.

**Table S1.** Geographical scale of subregions within the US, East Asia, and Europe

| Subregion | Elements |
| --- | --- |
| US_NENY | 'Connecticut', 'Maine', 'Massachusetts', 'New Hampshire', 'Rhode Island', 'Vermont', 'New York' |
| US_Mid_Atlantic | 'Delaware', 'Maryland', 'New Jersey', 'Ohio', 'Pennsylvania', 'West Virginia', 'Virginia' |
| US_Carolinas | 'North Carolina', 'South Carolina' |
| US_South | 'Alabama', 'US_Georgia', 'Florida' |
| US_Tennessee | 'Tennessee', 'Kentucky' |
| US_Midwest | 'Illinois','Indiana','Iowa','Michigan','Minnesota','Missouri','North Dakota', 'South Dakota', 'Wisconsin', 'Arkansas', 'Louisiana', 'Mississippi' |
| US_Central | 'Kansas', 'Nebraska', 'Oklahoma' |
| US_Texas | 'Texas' |
| US_Southwest | 'Arizona', 'New Mexico' |
| US_Northwest | 'Colorado','Idaho','Montana','Nevada','Oregon','Utah','Washington', 'Wyoming' |
| US_California | 'California' |
| CN_North | 'Beijing', 'Hebei', 'Inner Mongolia', 'Shandong', 'Shanxi', 'Tianjin' |
| CN_Northwest | 'Gansu', 'Ningxia', 'Qinghai', 'Shaanxi', 'Xinjiang' |
| CN_Northeast | 'Heilongjiang', 'Jilin', 'Liaoning' |
| CN_East | 'Anhui', 'Fujian', 'Jiangsu', 'Shanghai', 'Zhejiang' |
| CN_Central | 'Chongqing', 'Henan', 'Hubei', 'Hunan', 'Jiangxi', 'Sichuan' |
| CN_South | 'Guangdong', 'Guangxi', 'Guizhou', 'Hainan', 'Yunnan' |
| CN_Tibet | 'Tibet' |
| Japan_Korea | 'North Korea', 'South Korea', 'Japan' |
| Asia_Mongolia | 'Mongolia' |
| Iberia | 'Spain', 'Portugal' |
| British_Isles | 'United Kingdom', 'Ireland' |
| Europe_CW | 'Germany', 'Netherlands', 'Belgium', 'Luxembourg', 'Austria', 'Switzerland', 'France' |
| Nordic_Baltic | 'Denmark','Norway','Sweden','Finland','Estonia','Latvia','Lithuania','Iceland' |
| Europe_E | 'Poland', 'Czechia', 'Slovakia', 'Hungary', 'Romania', 'Bulgaria' |
| Europe_S | 'Italy', 'Slovenia', 'Croatia', 'Bosnia and Herz.','Serbia','Greece','Albania','Macedonia','Kosovo','Montenegro' |

Note: 1) An element at the lowest level (either country, province, or state’) are marked by ‘quotation marks’, whose meteorological reanalysis data is downloading based on its shapefile provided by ‘Natural Earth Dataset’ or GADM. 2) This study takes no position on any sovereign status and boundary delimitation.

**Table S4.** CV of solar power series from Level 1 to Level 2

| Level | Name | Cap  (GW) | avg | CV  (%) | ptp/avg | CF  (%) | Availability  (%) |
| --- | --- | --- | --- | --- | --- | --- | --- |
| 1 | United Kingdom | 1,187 | 157 | 145.56 | 6.50 | 13.22 | 52.24 |
| 1 | France | 2,687 | 472 | 135.82 | 5.13 | 17.58 | 53.80 |
| 1 | Germany | 1,715 | 245 | 144.74 | 6.18 | 14.26 | 52.17 |
| 1 | Poland | 1,503 | 200 | 146.15 | 6.40 | 13.32 | 52.24 |
| 1 | Romania | 1,129 | 186 | 140.37 | 5.39 | 16.45 | 51.93 |
| 1 | Italy | 1,444 | 282 | 131.65 | 4.50 | 19.54 | 52.68 |
| 1 | Sweden | 1,999 | 241 | 152.98 | 6.88 | 12.07 | 54.65 |
| 1 | Finland | 1,492 | 165 | 160.23 | 7.63 | 11.07 | 56.56 |
| 1 | Spain | 2,452 | 520 | 131.45 | 4.38 | 21.20 | 52.03 |
| 1 | Greece | 633 | 127 | 132.35 | 4.51 | 20.15 | 50.70 |
| 1 | Inner Mongolia | 5,622 | 1,201 | 129.65 | 4.44 | 21.36 | 55.65 |
| 1 | Xinjiang | 8,008 | 1,925 | 126.88 | 4.14 | 24.04 | 55.47 |
| 1 | Gansu | 2,090 | 478 | 129.84 | 4.32 | 22.85 | 52.71 |
| 1 | Jilin | 938 | 177 | 136.79 | 4.95 | 18.88 | 51.31 |
| 1 | Jiangsu | 523 | 92 | 140.96 | 5.28 | 17.53 | 50.16 |
| 1 | Henan | 813 | 147 | 138.21 | 5.18 | 18.07 | 50.76 |
| 1 | Guangdong | 882 | 144 | 137.30 | 5.25 | 16.32 | 51.17 |
| 1 | Shandong | 775 | 145 | 136.36 | 4.86 | 18.66 | 51.37 |
| 1 | Mongolia | 7,625 | 1,633 | 130.15 | 4.35 | 21.41 | 56.01 |
| 1 | Japan | 1,789 | 308 | 136.12 | 5.18 | 17.19 | 54.31 |
| 1 | New York | 599 | 101 | 142.11 | 5.68 | 16.90 | 51.27 |
| 1 | Virginia | 502 | 91 | 138.55 | 5.11 | 18.22 | 51.27 |
| 1 | North Carolina | 618 | 115 | 135.79 | 4.98 | 18.65 | 51.13 |
| 1 | Georgia | 731 | 140 | 135.41 | 4.94 | 19.11 | 50.33 |
| 1 | Tennessee | 524 | 97 | 136.74 | 5.04 | 18.44 | 51.77 |
| 1 | Texas | 3,321 | 752 | 129.22 | 4.09 | 22.66 | 51.84 |
| 1 | Kansas | 1,039 | 224 | 133.03 | 4.45 | 21.57 | 51.00 |
| 1 | North Dakota | 877 | 164 | 139.48 | 5.35 | 18.70 | 51.04 |
| 1 | Minnesota | 1,012 | 179 | 141.02 | 5.45 | 17.70 | 51.55 |
| 1 | Iowa | 710 | 133 | 139.70 | 5.07 | 18.77 | 50.39 |
| 2 | Europe_EU_plus | 24,293 | 3,773 | 129.32 | 4.80 | 15.53 | 73.30 |
| 2 | Asia_E | 57,002 | 11,917 | 120.29 | 4.12 | 20.91 | 69.71 |
| 2 | US_East | 22,637 | 4,377 | 125.05 | 4.40 | 19.34 | 60.92 |

Note: 1-Country/Province/State, 2-Regional, 3-Continental, 4-Inter-Continental, 5-Global.

**Table S3.** Elements in Russia

| Elements in Russia | Federal subjects |
| --- | --- |
| 'RU_Siberian_W' | Altay, Gorno-Altay, Kemerovo, Novosibirsk, Omsk, Tomsk, Khakass, |
| 'RU_Siberian_E' | Irkutsk, Tuva, Buryat |
| 'RU_Siberian_N' | Krasnoyarsk |
| 'RU_FarEastern_NW' | Sakha |
| 'RU_FarEastern_L', | Amur, Yevrey, Zabaykal’ye, Primor’ye, Sakhalin, Khabarovsk |
| 'RU_FarEastern_U' | Kamchatka, Maga, Buryatdan, Chukot |
| 'RU_Central' | Belgorod, Bryansk, Vladimir, Voronezh, Ivanovo, Kaluga, Kostroma, Kursk, Lipetsk, Moscow City, Moskva, Orel, Ryazan’, Smolensk, Tambov, Tver’, Tula, Yaroslav’ |
| 'RU_Volga' | Bashkortostan, Kirov, Mariy-El, Mordovia, Nizhegorod, Orenburg, Penza, Perm’, Samara, Saratov, Tatarstan, Udmurt, Ul’yanovsk, Chuvash' |
| 'RU_Northwestern_E' | Arkhangel’sk, Vologda, Komi, Nenets |
| 'RU_Northwestern_W' | Karelia, Leningrad, Murmansk, Novgorod, Pskov |
| 'RU_Southern_Caucasian' | Adygey, Astrakhan, Kalmyk, Krasnodar, Rostov, Volgograd, Dagestan, Ingush, Kabardin-Balkar, Karachay-Cherkess, North Ossetia, Stavropol’, Chechnya, |
| 'RU_Ural_N' | Yamal-Nenets |
| 'RU_Ural_S' | Kurgan, Sverdlovsk, Tyumen’, Khanty-Mansiy, Chelyabinsk, |

**Table S5.** CV of solar power series from Level 2 to Level 5

| Level | Name | Cap  (GW) | avg | CV  (%) | ptp/avg | CF  (%) | Availability  (%) |
| --- | --- | --- | --- | --- | --- | --- | --- |
| 2 | Europe_EU_plus | 24,293 | 3,773 | 129.32 | 4.80 | 15.53 | 73.30 |
| 2 | Europe_RU_plus | 24,113 | 3,275 | 137.66 | 5.59 | 13.58 | 66.56 |
| 2 | Asia_E | 57,002 | 11,917 | 120.29 | 4.12 | 20.91 | 69.71 |
| 2 | Asia_C | 22,471 | 4,615 | 128.89 | 4.30 | 20.54 | 59.55 |
| 2 | Asia_S | 20,213 | 4,475 | 125.77 | 4.13 | 22.14 | 59.32 |
| 2 | Asia_SE | 23,522 | 4,456 | 121.84 | 3.83 | 18.94 | 67.51 |
| 2 | Asia_W | 30,579 | 7,333 | 122.30 | 3.71 | 23.98 | 59.37 |
| 2 | Asia_RU | 64,278 | 8,900 | 118.85 | 4.68 | 13.85 | 81.87 |
| 2 | US_East | 22,637 | 4,377 | 125.05 | 4.40 | 19.34 | 60.92 |
| 2 | US_West | 14,923 | 3,394 | 126.82 | 4.09 | 22.74 | 55.59 |
| 2 | CA_East | 13,680 | 2,085 | 133.69 | 6.02 | 15.24 | 61.87 |
| 2 | CA_West | 13,314 | 2,246 | 133.36 | 5.15 | 16.87 | 63.05 |
| 2 | N_America_High | 24,893 | 3,296 | 133.64 | 6.07 | 13.24 | 73.98 |
| 2 | N_America_MX | 9,541 | 2,307 | 124.26 | 3.69 | 24.18 | 54.03 |
| 2 | South_America_N | 27,229 | 5,725 | 124.37 | 3.76 | 21.03 | 60.88 |
| 2 | South_America_E | 41,022 | 8,391 | 126.14 | 3.87 | 20.46 | 57.94 |
| 2 | South_America_S | 19,890 | 4,557 | 126.57 | 3.89 | 22.91 | 56.35 |
| 2 | Africa_N | 28,164 | 7,087 | 118.82 | 3.46 | 25.16 | 63.91 |
| 2 | Africa_SE | 69,392 | 15,844 | 122.90 | 3.52 | 22.83 | 59.94 |
| 2 | Africa_CW | 44,377 | 10,468 | 119.45 | 3.44 | 23.59 | 61.15 |
| 3 | Europe | 48,406 | 7,048 | 125.76 | 4.64 | 14.56 | 77.82 |
| 3 | Asia | 218,066 | 41,696 | 105.65 | 3.51 | 19.12 | 91.03 |
| 3 | North_America | 98,987 | 17,703 | 118.52 | 4.18 | 17.88 | 81.94 |
| 3 | South_America | 88,141 | 18,673 | 120.77 | 3.66 | 21.19 | 65.98 |
| 3 | Africa | 141,933 | 33,399 | 116.17 | 3.34 | 23.53 | 68.44 |
| 3 | Oceania | 37,752 | 8,639 | 123.89 | 3.71 | 22.88 | 58.74 |
| 4 | Europe-Asia | 266,472 | 48,744 | 97.73 | 3.28 | 18.29 | 96.90 |
| 4 | Europe-Africa | 190,338 | 40,447 | 115.59 | 3.44 | 21.25 | 80.60 |
| 4 | Europe-North_America | 147,393 | 24,752 | 76.82 | 2.99 | 16.79 | 98.59 |
| 4 | Asia-Africa | 359,998 | 75,094 | 89.56 | 2.93 | 20.86 | 98.70 |
| 4 | Asia-North_America | 317,053 | 59,399 | 56.11 | 2.42 | 18.73 | 100.00 |
| 4 | North-South_America | 187,128 | 36,377 | 98.60 | 3.18 | 19.44 | 87.06 |
| 4 | Europe-Asia-Africa | 408,404 | 82,142 | 89.91 | 2.90 | 20.11 | 98.70 |
| 4 | Europe-Asia-North_America | 365,459 | 66,447 | 53.90 | 2.32 | 18.18 | 100.00 |
| 5 | Global | 633,285 | 127,158 | 43.50 | 1.76 | 20.08 | 100.00 |

Note: 1-Country/Province/State, 2-Regional, 3-Continental, 4-Inter-Continental, 5-Global.

**Table S6.** CV of solar power series globally at Level 1 as well as reductions in regional and continental aggregation (Part I)

| Level | Name | CV (%) | Reduc-  tion1 | Reduc-  tion2 | Name | CV (%) | Reduc-  tion1 | Reduc-  tion2 |
| --- | --- | --- | --- | --- | --- | --- | --- | --- |
| 1 | Spain | 131.45 | 2.13 | 5.69 | Anhui | 141.76 | 21.47 | 36.11 |
| 1 | Portugal | 133.94 | 4.62 | 8.18 | Fujian | 140.46 | 20.17 | 34.81 |
| 1 | United Kingdom | 145.56 | 16.23 | 19.80 | Jiangsu | 140.96 | 20.67 | 35.31 |
| 1 | Ireland | 150.88 | 21.56 | 25.13 | Shanghai | 146.39 | 26.11 | 40.75 |
| 1 | Germany | 144.74 | 15.41 | 18.98 | Zhejiang | 144.78 | 24.49 | 39.13 |
| 1 | Netherlands | 151.51 | 22.19 | 25.75 | Chongqing | 149.30 | 29.01 | 43.65 |
| 1 | Belgium | 151.67 | 22.35 | 25.92 | Henan | 138.21 | 17.92 | 32.56 |
| 1 | Luxembourg | 155.57 | 26.24 | 29.81 | Hubei | 143.06 | 22.77 | 37.41 |
| 1 | Austria | 141.58 | 12.26 | 15.82 | Hunan | 146.76 | 26.47 | 41.12 |
| 1 | Switzerland | 140.08 | 10.76 | 14.32 | Jiangxi | 143.98 | 23.69 | 38.33 |
| 1 | France | 135.82 | 6.50 | 10.06 | Sichuan | 129.71 | 9.42 | 24.07 |
| 1 | Denmark | 153.29 | 23.97 | 27.54 | Guangdong | 137.30 | 17.01 | 31.65 |
| 1 | Norway | 149.35 | 20.03 | 23.59 | Guangxi | 139.29 | 19.00 | 33.64 |
| 1 | Sweden | 152.98 | 23.66 | 27.23 | Guizhou | 143.43 | 23.14 | 37.78 |
| 1 | Finland | 160.23 | 30.91 | 34.47 | Hainan | 133.60 | 13.31 | 27.95 |
| 1 | Estonia | 159.00 | 29.68 | 33.24 | Yunnan | 131.86 | 11.57 | 26.21 |
| 1 | Latvia | 156.17 | 26.85 | 30.41 | Tibet | 125.76 | 5.47 | 20.11 |
| 1 | Lithuania | 156.21 | 26.89 | 30.45 | Mongolia | 130.15 | 9.86 | 24.51 |
| 1 | Iceland | 158.94 | 29.62 | 33.18 | North Korea | 137.36 | 17.07 | 31.72 |
| 1 | Poland | 146.15 | 16.83 | 20.39 | South Korea | 137.83 | 17.54 | 32.18 |
| 1 | Czechia | 147.62 | 18.30 | 21.86 | Japan | 136.12 | 15.83 | 30.47 |
| 1 | Slovakia | 146.51 | 17.19 | 20.76 | Kazakhstan | 131.89 | 2.99 | 26.24 |
| 1 | Hungary | 144.27 | 14.95 | 18.52 | Uzbekistan | 131.98 | 3.08 | 26.33 |
| 1 | Romania | 140.37 | 11.05 | 14.61 | Tajikistan | 129.83 | 0.94 | 24.18 |
| 1 | Bulgaria | 137.63 | 8.31 | 11.87 | Kyrgyzstan | 130.77 | 1.88 | 25.12 |
| 1 | Italy | 131.65 | 2.33 | 5.90 | Turkmenistan | 131.46 | 2.57 | 25.81 |
| 1 | Slovenia | 145.18 | 15.86 | 19.42 | Afghanistan | 127.13 | -1.77 | 21.48 |
| 1 | Croatia | 138.13 | 8.81 | 12.38 | India | 127.60 | 1.83 | 21.95 |
| 1 | Bosnia and Herz. | 139.90 | 10.58 | 14.14 | Pakistan | 125.59 | -0.18 | 19.94 |
| 1 | Serbia | 141.04 | 11.72 | 15.29 | Bangladesh | 133.32 | 7.56 | 27.68 |
| 1 | Greece | 132.35 | 3.03 | 6.59 | Bhutan | 132.29 | 6.52 | 26.64 |
| 1 | Albania | 135.84 | 6.52 | 10.09 | Nepal | 130.67 | 4.90 | 25.02 |
| 1 | Macedonia | 137.80 | 8.48 | 12.04 | Sri Lanka | 129.30 | 3.54 | 23.66 |
| 1 | Kosovo | 139.68 | 10.36 | 13.93 | Cambodia | 129.47 | 7.63 | 23.83 |
| 1 | Montenegro | 140.14 | 10.81 | 14.38 | Thailand | 128.89 | 7.05 | 23.24 |
| 1 | RU_Central | 149.81 | 12.16 | 24.06 | Laos | 129.90 | 8.06 | 24.25 |
| 1 | RU_Volga | 144.79 | 7.14 | 19.04 | Myanmar | 132.12 | 10.28 | 26.47 |
| 1 | RU_Northwestern_E | 154.90 | 17.24 | 29.14 | Vietnam | 129.65 | 7.80 | 24.00 |
| 1 | RU_Northwestern_W | 156.79 | 19.14 | 31.03 | Philippines | 127.86 | 6.02 | 22.21 |
| 1 | RU_Southern_Caucasian | 135.82 | -1.84 | 10.06 | Malaysia | 130.11 | 8.27 | 24.47 |
| 1 | Ukraine | 142.60 | 4.94 | 16.84 | Indonesia | 124.70 | 2.85 | 19.05 |
| 1 | Belarus | 151.75 | 14.10 | 26.00 | Iran | 125.56 | 3.27 | 19.92 |
| 1 | Georgia | 132.65 | -5.01 | 6.89 | Iraq | 126.49 | 4.20 | 20.85 |
| 1 | Gansu | 129.84 | 9.55 | 24.19 | Israel | 127.87 | 5.57 | 22.22 |
| 1 | Ningxia | 134.37 | 14.08 | 28.72 | Jordan | 126.60 | 4.30 | 20.95 |
| 1 | Qinghai | 127.95 | 7.66 | 22.30 | Kuwait | 127.55 | 5.25 | 21.90 |
| 1 | Shaanxi | 136.19 | 15.90 | 30.54 | Lebanon | 130.04 | 7.74 | 24.39 |
| 1 | Xinjiang | 126.88 | 6.59 | 21.23 | Oman | 125.58 | 3.29 | 19.94 |
| 1 | Beijing | 138.20 | 17.91 | 32.55 | Qatar | 126.19 | 3.90 | 20.55 |
| 1 | Hebei | 135.29 | 15.00 | 29.64 | Saudi Arabia | 123.20 | 0.90 | 17.56 |
| 1 | Inner Mongolia | 129.65 | 9.37 | 24.01 | Syria | 127.89 | 5.60 | 22.25 |
| 1 | Shandong | 136.36 | 16.07 | 30.71 | United Arab Emirates | 125.84 | 3.54 | 20.19 |
| 1 | Shanxi | 135.59 | 15.30 | 29.95 | Yemen | 124.53 | 2.24 | 18.89 |
| 1 | Tianjin | 138.09 | 17.80 | 32.45 | Turkey | 129.46 | 7.16 | 23.81 |
| 1 | Heilongjiang | 135.97 | 15.68 | 30.33 | Palestine | 129.46 | 7.16 | 23.81 |
| 1 | Jilin | 136.79 | 16.50 | 31.14 | RU_Ural_N | 156.62 | 37.77 | 50.98 |
| 1 | Liaoning | 135.85 | 15.56 | 30.21 | RU_Ural_S | 146.47 | 27.62 | 40.83 |

Note: 1) 1-Country/Province/State, 2-Regional, 3-Continental, 4-Inter-Continental, 5-Global. 2) ‘Reduction 1’ is calculated by the CV of ‘Region’ minus that of ‘Country’, e.g., the CV of ‘Spain’ is 131.45% in Table S4 and the CV of corresponding region ‘Europe_EU_plus’ is 129.32% in Table S5, thus the difference is 2.13%. 3) ‘Reduction 2’ is calculated by the CV of ‘Continent’ minus that of ‘Country’. 4) The CV of regional aggregation will increase for only five countries in Table S6 and Table S7, accounting for 2.2% (5 out of 229).

**Table S7.** CV of solar power series globally at Level 1 as well as reductions in regional and continental aggregation (Part II)

| Level | Name | CV (%) | Reduc-  tion1 | Reduc-  tion2 | Name | CV (%) | Reduc-  tion1 | Reduc-  tion2 |
| --- | --- | --- | --- | --- | --- | --- | --- | --- |
| 1 | RU_Siberian_W | 140.10 | 21.25 | 34.45 | Prince Edward Island | 151.35 | 17.67 | 32.83 |
| 1 | RU_Siberian_E | 133.94 | 15.09 | 28.29 | Nova Scotia | 150.84 | 17.15 | 32.32 |
| 1 | RU_Siberian_N | 145.49 | 26.64 | 39.84 | Newfoundland and Labrador | 144.22 | 10.53 | 25.70 |
| 1 | RU_FarEastern_NW | 142.05 | 23.20 | 36.41 | Manitoba | 142.94 | 9.58 | 24.42 |
| 1 | RU_FarEastern_L | 131.18 | 12.33 | 25.54 | Saskatchewan | 139.67 | 6.31 | 21.15 |
| 1 | RU_FarEastern_U | 143.23 | 24.38 | 37.58 | Alberta | 138.36 | 5.00 | 19.83 |
| 1 | Connecticut | 145.01 | 19.96 | 26.49 | British Columbia | 139.47 | 6.11 | 20.95 |
| 1 | Maine | 146.91 | 21.86 | 28.39 | USA_Alaska | 148.25 | 14.61 | 29.73 |
| 1 | Massachusetts | 144.11 | 19.06 | 25.59 | CA_Yukon | 146.67 | 13.03 | 28.15 |
| 1 | New Hampshire | 147.05 | 22.00 | 28.53 | CA_Northwest | 148.85 | 15.21 | 30.33 |
| 1 | Rhode Island | 145.56 | 20.50 | 27.03 | CA_Nunavut | 150.08 | 16.43 | 31.55 |
| 1 | Vermont | 147.10 | 22.05 | 28.58 | Mexico | 124.26 | - | 5.74 |
| 1 | New York | 142.11 | 17.06 | 23.59 | Guatemala | 127.97 | 3.60 | 7.21 |
| 1 | Delaware | 142.18 | 17.13 | 23.66 | Honduras | 127.12 | 2.75 | 6.35 |
| 1 | Maryland | 141.63 | 16.58 | 23.11 | Panama | 130.68 | 6.31 | 9.91 |
| 1 | New Jersey | 142.88 | 17.83 | 24.36 | Colombia | 127.97 | 3.60 | 7.20 |
| 1 | Ohio | 141.35 | 16.30 | 22.82 | Venezuela | 126.48 | 2.11 | 5.71 |
| 1 | Pennsylvania | 142.39 | 17.34 | 23.87 | Guyana | 128.74 | 4.37 | 7.97 |
| 1 | West Virginia | 141.21 | 16.16 | 22.69 | Guyane | 131.28 | 6.91 | 10.51 |
| 1 | Virginia | 138.55 | 13.50 | 20.03 | Suriname | 130.46 | 6.09 | 9.70 |
| 1 | North Carolina | 135.79 | 10.74 | 17.27 | Ecuador | 129.08 | 4.71 | 8.31 |
| 1 | South Carolina | 136.41 | 11.35 | 17.88 | Peru | 127.36 | 2.99 | 6.59 |
| 1 | Alabama | 136.68 | 11.63 | 18.16 | Bolivia | 126.23 | 1.86 | 5.46 |
| 1 | US_Georgia | 135.41 | 10.36 | 16.89 | Brazil | 126.14 | 0.00 | 5.37 |
| 1 | Florida | 129.31 | 4.26 | 10.79 | Chile | 125.31 | -1.26 | 4.54 |
| 1 | Tennessee | 136.74 | 11.69 | 18.22 | Argentina | 127.81 | 1.25 | 7.05 |
| 1 | Kentucky | 140.25 | 15.20 | 21.73 | Uruguay | 138.27 | 11.70 | 17.50 |
| 1 | Illinois | 139.71 | 14.66 | 21.19 | Paraguay | 133.53 | 6.96 | 12.76 |
| 1 | Indiana | 140.80 | 15.75 | 22.28 | Egypt | 123.92 | 5.10 | 7.75 |
| 1 | Iowa | 139.70 | 14.65 | 21.17 | Libya | 123.64 | 4.82 | 7.47 |
| 1 | Michigan | 140.85 | 15.80 | 22.33 | Algeria | 123.26 | 4.44 | 7.09 |
| 1 | Minnesota | 141.02 | 15.97 | 22.50 | Morocco | 124.48 | 5.66 | 8.31 |
| 1 | Missouri | 137.36 | 12.30 | 18.83 | Tunisia | 127.11 | 8.29 | 10.94 |
| 1 | North Dakota | 139.48 | 14.42 | 20.95 | Dem. Rep. Congo | 126.64 | 3.74 | 10.47 |
| 1 | South Dakota | 135.65 | 10.60 | 17.13 | Angola | 126.77 | 3.87 | 10.60 |
| 1 | Wisconsin | 142.13 | 17.07 | 23.60 | Zimbabwe | 128.18 | 5.28 | 12.01 |
| 1 | Arkansas | 137.25 | 12.19 | 18.72 | Botswana | 126.47 | 3.57 | 10.30 |
| 1 | Louisiana | 133.09 | 8.03 | 14.56 | Namibia | 125.22 | 2.32 | 9.05 |
| 1 | Mississippi | 136.54 | 11.49 | 18.02 | Zambia | 127.04 | 4.14 | 10.87 |
| 1 | Kansas | 133.03 | 7.98 | 14.51 | Mozambique | 126.90 | 4.00 | 10.73 |
| 1 | Nebraska | 134.19 | 9.13 | 15.66 | South Africa | 126.66 | 3.76 | 10.49 |
| 1 | Oklahoma | 132.50 | 7.45 | 13.98 | Tanzania | 126.66 | 3.76 | 10.49 |
| 1 | Texas | 129.22 | 4.17 | 10.69 | Somalia | 127.14 | 4.24 | 10.97 |
| 1 | Arizona | 127.52 | 0.70 | 9.00 | Sudan | 123.43 | 0.53 | 7.26 |
| 1 | New Mexico | 127.77 | 0.95 | 9.25 | S. Sudan | 126.10 | 3.20 | 9.93 |
| 1 | Colorado | 129.97 | 3.15 | 11.45 | Ethiopia | 125.74 | 2.84 | 9.57 |
| 1 | Idaho | 133.53 | 6.70 | 15.00 | Kenya | 125.69 | 2.79 | 9.52 |
| 1 | Montana | 134.56 | 7.74 | 16.04 | Chad | 123.21 | 3.76 | 7.04 |
| 1 | Nevada | 129.45 | 2.63 | 10.93 | Cameroon | 127.04 | 7.59 | 10.87 |
| 1 | Oregon | 135.56 | 8.74 | 17.04 | Congo | 129.28 | 9.83 | 13.11 |
| 1 | Utah | 129.50 | 2.68 | 10.98 | Central African Rep. | 126.77 | 7.32 | 10.60 |
| 1 | Washington | 139.98 | 13.16 | 21.46 | Mali | 123.93 | 4.48 | 7.76 |
| 1 | Wyoming | 132.29 | 5.46 | 13.76 | Mauritania | 124.05 | 4.60 | 7.88 |
| 1 | California | 128.17 | 1.35 | 9.65 | Niger | 123.68 | 4.23 | 7.51 |
| 1 | Ontario | 137.71 | 4.03 | 19.19 | Nigeria | 125.28 | 5.83 | 9.11 |
| 1 | Quebec | 140.62 | 6.93 | 22.09 | Guinea | 125.57 | 6.12 | 9.40 |
| 1 | New Brunswick | 149.60 | 15.91 | 31.08 | Ghana | 125.58 | 6.13 | 9.41 |
|  |  |  |  |  | Australia | 123.89 | - | - |

Note: 1) 1-Country/Province/State, 2-Regional, 3-Continental, 4-Inter-Continental, 5-Global. 2) ‘Reduction 1’ is calculated by the CV of ‘Region’ minus that of ‘Country’, e.g., the CV of ‘Spain’ is 131.45% in Table S4 and the CV of corresponding region ‘Europe_EU_plus’ is 129.32% in Table S5, thus the difference is 2.13%. 3) ‘Reduction 2’ is calculated by the CV of ‘Continent’ minus that of ‘Country’. 4) ‘Australia’ is the only country considered for continent ‘Oceania’, thus there are no regional and continental reduction. 5) The CV of regional aggregation will increase for only five countries in Table S6 and Table S7, accounting for 2.2% (5 out of 229).


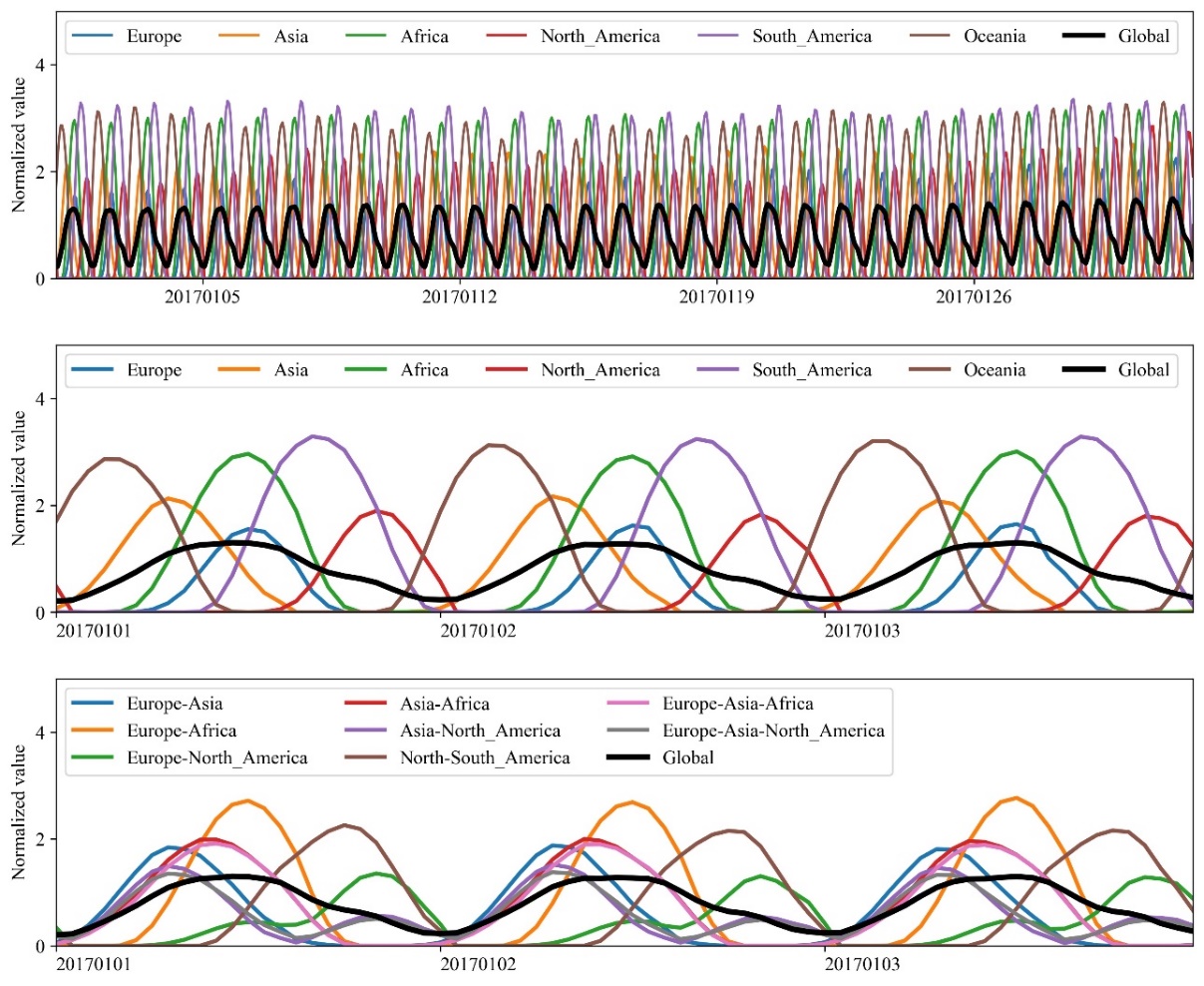


**Figure S2.** Solar power series of continental and inter-continental aggregation

**(a).** Solar power (July 2017) for regions in continental aggregation

**(b).** Solar power (1^st^ ~ 3^rd^ July 2017) for continental aggregation

**(c).** Solar power (1^st^ ~ 3^rd^ January 2017) for inter-continent aggregation


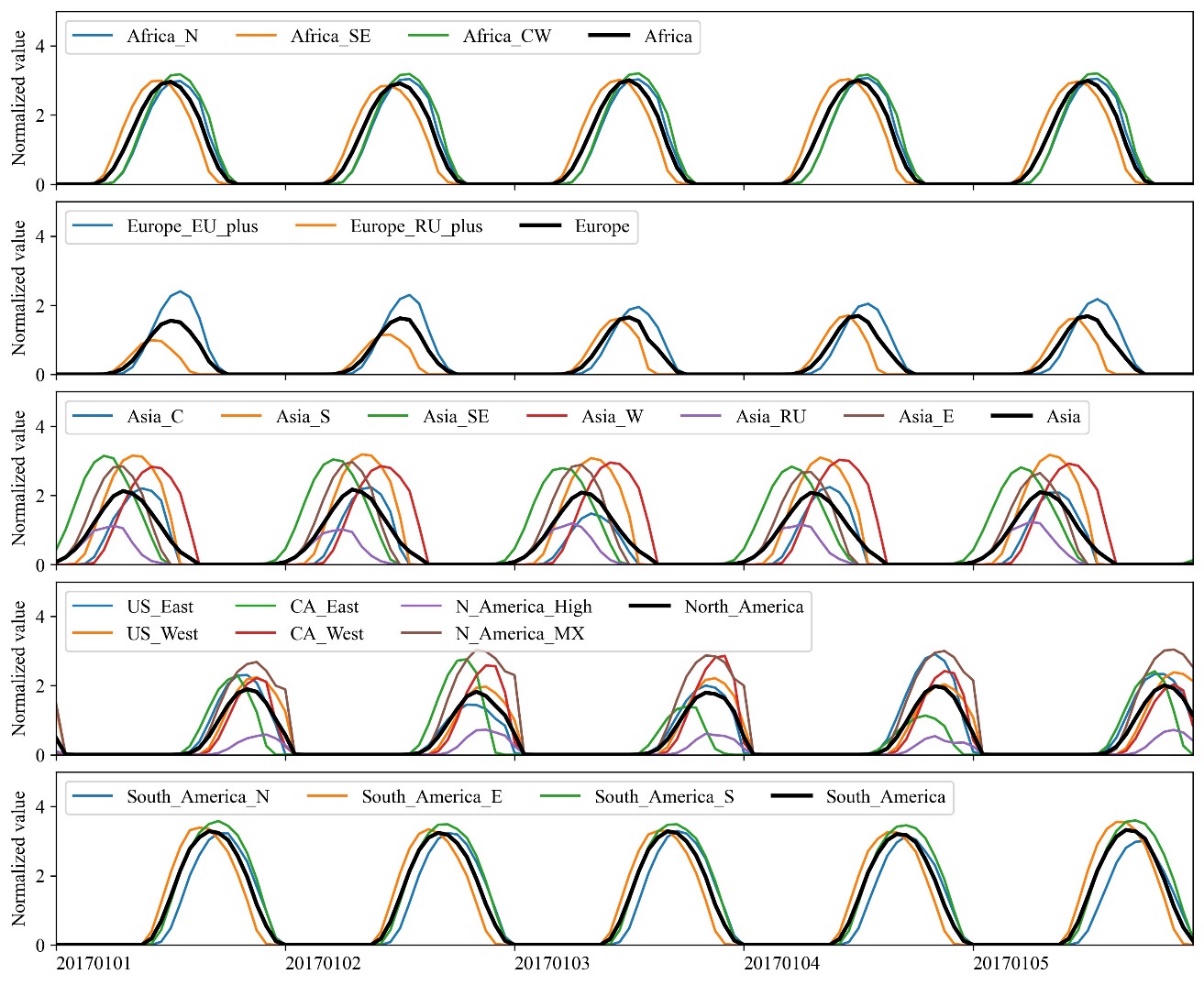


**Figure S1.** Solar power (1^st^ ~ 5^th^ January 2017) of regions in each continent
